# Complete Genome Sequences of Three Ebola Virus Isolates from the 2014 Outbreak in West Africa

**DOI:** 10.1128/genomeA.01331-14

**Published:** 2014-12-18

**Authors:** T. Hoenen, A. Groseth, F. Feldmann, A. Marzi, H. Ebihara, G. Kobinger, S. Günther, H. Feldmann

**Affiliations:** a Laboratory of Virology, Division of Intramural Research, National Institute of Allergy and Infectious Diseases, National Institutes of Health, Hamilton, Montana, USA; b Rocky Mountain Veterinary Branch, Division of Intramural Research, National Institute of Allergy and Infectious Diseases, National Institutes of Health, Hamilton, Montana, USA; c Special Pathogens Program, Public Health Agency of Canada, Winnipeg, Manitoba, Canada; d Bernard Nocht Institute for Tropical Medicine, Hamburg, Germany

## Abstract

Here, we report the complete genome sequences, including the genome termini, of three Ebola virus isolates (species *Zaire ebolavirus*) originating from Guinea that are now being widely used in laboratories in North America for research regarding West African Ebola viruses.

## GENOME ANNOUNCEMENT

Ebola virus (EBOV) causes a severe hemorrhagic fever with case fatality rates of up to 90% (1). Currently, no vaccines or therapeutics are approved for use in humans, although a number of promising experimental vaccines and treatment options exist (2). In the past, EBOV outbreaks have been restricted to central Africa (3); however, in December 2013, EBOV emerged in West Africa in the province of Gueckedou in Guinea (4). Since then, it has spread throughout Guinea and into the neighboring countries of Sierra Leone and Liberia, and imported cases have occurred in Senegal, Nigeria, and Mali. This outbreak, which is still ongoing, has affected more people and resulted in a greater number of deaths than all previous outbreaks combined (13,268 cases and 4,960 deaths as of 7 November 2014; http://apps.who.int/iris/bitstream/10665/137592/1/roadmapsitrep_7Nov2014_eng.pdf), and an unprecedented effort by the international community will be necessary to contain it.

We have determined the complete genome sequences, including the genome termini, of three virus isolates originating from Guinea (Ebola virus/*H. sapiens*-tc/GIN/2014/Makona-WPGC05, Ebola virus/*H. sapiens*-tc/GIN/2014/Makona-WPGC07, and Ebola virus/*H. sapiens*-tc/GIN/2014/Makona-WPGC15). These viruses were grown in Vero E6 cells (sequences were derived from p1 stocks) and are now used by a number of high-containment laboratories in North America, making their sequences of interest to these facilities. RNA was extracted from cell supernatant using a viral RNA extraction kit (Qiagen) using protocols approved by the Institutional Biosafety Committee and reverse transcribed using Superscript III (Invitrogen). PCR products of approximately 700 bp in length were amplified using IProof polymerase (Bio-rad) and subjected to conventional Sanger sequencing. Terminal sequences were determined by 5′ and 3′ rapid amplification of cDNA ends (RACE) based on ligation-anchored PCR, as previously described (5–7). Sequences were assembled using SeqMan Pro (Lasergene; DNAStar), and fragment ends were trimmed to exclude primer sequences.

All three full-length genome sequences showed very high similarities to nearly complete genome sequences of West African EBOVs previously published, although there were between 2 and 4 nucleotide differences to published EBOV sequences from Guinea (4) and between 7 and 17 nucleotide differences to published EBOV sequences from Sierra Leone (8). Surprisingly, in addition, the 5′ end of the genomic RNA was very clearly 1 nucleotide shorter than previously reported EBOV genomes, lacking a previously reported terminal U residue. It should be noted that these are the first sequences from the West African EBOV outbreak for which the genome ends were experimentally determined. Sequencing of PCR fragments spanning the 3′ genome end showed two PCR product species, a predominant one in which the 3′ end matched previously published sequences (3′-GCC… sequence in negative orientation) and a minority species that was 1 nucleotide shorter (3′-CC…). These differences in the genome termini compared to previously published sequences might have implications for the mechanism of virus genome replication and warrant further studies.

### Nucleotide sequence accession numbers.

The complete genome sequences of the EBOV isolates WPGC05, WPGC07, and WPGC15 have been submitted to GenBank under the accession numbers KP096420, KP096421, and KP096422.

## References

[1] Hoenen T Groseth A Falzarano D Feldmann H 2006 Ebola virus: unravelling pathogenesis to combat a deadly disease. Trends Mol. Med. 12:206–215. 10.1016/j.molmed.2006.03.006. 16616875

[2] Falzarano D Feldmann H 2014 Possible leap ahead in filovirus therapeutics. Cell Res. 24:647–648. 10.1038/cr.2014.49. 24732011 PMC4042172

[3] Groseth A Feldmann H Strong JE 2007 The ecology of Ebola virus. Trends Microbiol. 15:408–416. 10.1016/j.tim.2007.08.001. 17698361

[4] Baize S Pannetier D Oestereich L Rieger T Koivogui L Magassouba N Soropogui B Sow MS Keita S De Clerck H Tiffany A Dominguez G Loua M Traore A Kolie M Malano ER Heleze E Bocquin A Mely S Raoul H Caro V Cadar D Gabriel M Pahlmann M Tappe D Schmidt-Chanasit J Impouma B Diallo AK Formenty P Van Herp M Günther S 2014 Emergence of Zaire Ebola virus disease in Guinea—preliminary report. N. Engl. J. Med. 371:1418–1425. 10.1056/NEJMoa1404505. 24738640

[5] Li Z Yu M Zhang H Wang HY Wang LF 2005 Improved rapid amplification of cDNA ends (RACE) for mapping both the 5′ and 3′ terminal sequences of paramyxovirus genomes. J. Virol. Methods 130:154–156. 10.1016/j.jviromet.2005.06.022. 16076500

[6] Tillett D Burns BP Neilan BA 2000 Optimized rapid amplification of cDNA ends (RACE) for mapping bacterial mRNA transcripts. Biotechniques 28:448–456. 10723556 10.2144/00283st01

[7] Troutt AB McHeyzer-Williams MG Pulendran B Nossal GJ 1992 Ligation-anchored PCR: a simple amplification technique with single-sided specificity. Proc. Natl. Acad. Sci. U. S. A. 89:9823–9825. 10.1073/pnas.89.20.9823. 1409706 PMC50225

[8] Gire SK Goba A Andersen KG Sealfon RSG Park DJ Kanneh L Jalloh S Momoh M Fullah M Dudas G Wohl S Moses LM Yozwiak NL Winnicki S Matranga CB Malboeuf CM Qu J Gladden AD Schaffner SF Yang X Jiang P-P Nekoui M Colubri A Coomber MR Fonnie M Moigboi A Gbakie M Kamara FK Tucker V Konuwa E Saffa S Sellu J Jalloh AA Kovoma A Koninga J Mustapha I Kargbo K Foday M Yillah M Kanneh F Robert W Massally JLB Chapman SB Bochicchio J Murphy C Nusbaum C Young S Birren BW Grant DS Scheiffelin JS Lander ES Happi C Gevao SM Gnirke A Rambaut A Garry RF Khan SH Sabeti PC 2014 Genomic surveillance elucidates Ebola virus origin and transmission during the 2014 outbreak. Science 345:1369–1372. 10.1126/science.1259657. 25214632 PMC4431643

